# Antitumor Activity against A549 Cancer Cells of Three Novel Complexes Supported by Coating with Silver Nanoparticles

**DOI:** 10.3390/ijms23062980

**Published:** 2022-03-10

**Authors:** Agnieszka Czylkowska, Bartłomiej Rogalewicz, Małgorzata Szczesio, Anita Raducka, Katarzyna Gobis, Paweł Szymański, Kamila Czarnecka, Bruno Cury Camargo, Jacek Szczytko, Alexey Babich, Sergey Dubkov, Petr Lazarenko

**Affiliations:** 1 Institute of General and Ecological Chemistry, Faculty of Chemistry, Lodz University of Technology, Zeromskiego 116, 90-924 Lodz, Poland; bartlomiej.rogalewicz@dokt.p.lodz.pl (B.R.); malgorzata.szczesio@p.lodz.pl (M.S.); anita.raducka@edu.p.lodz.pl (A.R.); 2 Department of Organic Chemistry, Faculty of Pharmacy, Medical University of Gdansk, Gen. Hallera 107, 80-416 Gdansk, Poland; katarzyna.gobis@gumed.edu.pl; 3 Department of Pharmaceutical Chemistry, Drug Analyses and Radiopharmacy, Faculty of Pharmacy, Medical University of Lodz, Muszynskiego 1, 90-151 Lodz, Poland; pawel.szymanski@umed.lodz.pl (P.S.); kamila.czarnecka@umed.lodz.pl (K.C.); 4 Department of Radiobiology and Radiation Protection, Military Institute of Hygiene and Epidemiology, 4 Kozielska St., 01-163 Warsaw, Poland; 5 Institute of Experimental Physics, Faculty of Physics, University of Warsaw, Pasteura 5, 02-093 Warszawa, Poland; bruno.camargo@fuw.edu.pl (B.C.C.); jacek.szczytko@fuw.edu.pl (J.S.); 6 Institute of Advanced Materials and Technologies, National Research University of Electronic Technology, Bld. 1, Shokin Square, Zelenograd, 124498 Moscow, Russia; drent@yandex.ru (A.B.); sv.dubkov@gmail.com (S.D.); aka.jum@gmail.com (P.L.)

**Keywords:** antitumor activity, A549 lung cancer cells, coordination compound, FTIR spectroscopy, UV-VIS-NIR spectrophotometry, TG-DTG analysis, magnetization measurements

## Abstract

A novel biologically active organic ligand L (N’-benzylidenepyrazine-2-carbohydrazonamide) and its three coordination compounds have been synthesized and structurally described. Their physicochemical and biological properties have been thoroughly studied. Cu(II), Zn(II), and Cd(II) complexes have been analyzed by F-AAS spectrometry and elemental analysis. The way of metal–ligand coordination was discussed based on FTIR spectroscopy and UV-VIS-NIR spectrophotometry. The thermal behavior of investigated compounds was studied in the temperature range 25–800 °C. All compounds are stable at room temperature. The complexes decompose in several stages. Magnetic studies revealed strong antiferromagnetic interaction. Their cytotoxic activity against A549 lung cancer cells have been studied with promising results. We have also investigated the biological effect of coating studied complexes with silver nanoparticles. The morphology of the surface was studied using SEM imaging.

## 1. Introduction

Cancer is a general health problem worldwide. Lung cancer is the second most commonly diagnosed cancer in the United States and is a foremost cause of cancer deaths; thus, it is a major health problem [1]. The effectiveness of available treatments for lung cancer depends on the time of diagnosis. There are two main types of lung cancer: small-cell lung cancer (SCLC) and non-small-cell lung cancer (NSCLC) but approximately 75% are NSCLC. Approximately 80–90% of lung cancers are associated with smoking [2]. 

The use of pharmacologically active ligands in coordination compounds with the appropriate metals is one of the most promising strategies to find the right drug. One of the best examples is aromatic amine and imine derivatives. These compounds are at the center of interest due to their numerous pharmacological possibilities such as anticancer, antiviral, antimicrobial, antioxidant, and antineoplastic [3,4,5,6,7,8,9,10,11,12]. Coordination compounds containing such derivatives are considered to be a promising class of antineoplastic agent showing cytotoxic activity against different cell lines [13,14,15,16]. 

Another approach to enhance biological activity or modify physicochemical properties is coating compounds of a potential drug with metal nanoparticles. Nanoparticles are widely used due to their size and quantum-size effects, which lead to the appearance of unusual physicochemical, electrophysical, and optical properties. Recently, a lot of attention has been paid to arrays of noble metal nanoparticles, which are used in electronic devices and sensors [17,18,19,20]. Among a wide range of applications, a special role is assigned to the medical field, where gold and silver particles are already used in the delivery of therapeutic reagents [21], in the diagnosis of diseases [22], in the creation of antibacterial coatings [23], and also as a component of a sensitizer in the treatment of oncological diseases [24] by the method of photodynamic therapy, etc. 

Complexation of compounds containing donor nitrogen atoms with various metal ions such as Cu^2+^, Zn^2+^, or Cd^2+^ usually results in an increase in their biological activity [25,26]. It is well-known that compounds with antitumor activity based on endogenous metals such as those mentioned above would be more rational when it comes to a living organism. A key problem in current cancer therapy is the lack of neoplastic cell specificity. It leads to cytotoxicity against tumors as well as to normal cells and, consequently, causes drug resistance. The use of nanoparticles may be a solution in this case [27,28,29,30]. Development of carriers that enable drug delivery in an effective concentration to pathologically changed places without affecting normal cells is an appropriate method of targeted chemotherapy [31,32]. Drug encapsulation would also solve the problem of pharmaceutical solubility and distribution to specific sites in the body. 

Among all types of cancer, lung cancer is the leading cause of morbidity and mortality. It accounts for nearly 20% of cancer deaths in 2018. This cancer is the biggest problem of all, so we decided to use the A549 cell line for our research [33]. Therefore, three new coordination compounds were designed, synthesized, and investigated. The cytotoxic activity of the synthesized complexes was studied in vitro towards lung carcinoma cell line. The obtained IC_50_ values for the free ligand and its three coordination compounds (before and after coating with silver nanoparticles) have been compared with the IC_50_ of the cisplatin. The possible mechanism of action was also discussed.

## 2. Results and Discussion

### 2.1. Synthesis

#### 2.1.1. Synthesis of the Compound L

**Scheme 1:**
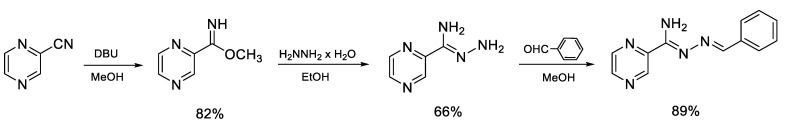
Synthesis route of the compound L.

Cyanopyrazine was converted into methyl pyrazine-2-carbimidate by treatment with methanol and catalytic amounts of DBU. Carbimidate in the reaction with hydrazine hydrate gave pyrazine-2-carbohydrazonamide. Condensation of carbohydrazonamide with benzaldehyde led to the corresponding imine compound L (Scheme 1).

##### Synthesis of Methyl Pyrazine-2-carbimidate

A total 11 mL (0.12 mol) of cyanopyrazine was dissolved in 30 mL of MeOH and 0.5 mL (3.3 mmol) of DBU (1,8-diazabicyclo[5.4.0]undec-7-ene) was added. The mixture was refluxed for 1 h, cooled, and the carbimidate precipitate was filtered off. A total 13.5 g (82%) of the product was obtained. Carbimidate was recrystallized from methanol. Analytical data were in accordance with literature [34].

##### Synthesis of Pyrazine-2-carbohydrazonamide

We suspended 1.37 g (10 mmol) of the carbimidate in 5 mL of EtOH and 1 mL of (32 mmol) 80% hydrazine hydrate was added. The mixture was refluxed for 15 min and then cooled. The carbohydrazonamide was isolated by filtration. A total 0.905 g (66%) of the product was obtained. Carbohydrazonamide was recrystallized from ethanol. Analytical data were in accordance with literature [35].

##### Synthesis of *N*′-Benzylidenepyrazine-2-carbohydrazonamide

We dissolved 0.685 g (5 mmol) of carbohydrazonamide in 15 mL of MeOH while hot and 0.510 mL (5 mmol) of benzaldehyde was then added. The mixture was left at room temperature for 0.5 h, then cooled, and the resulting imine was filtered off. A total 0.996 g (89%) of the product was obtained. The product was recrystallized from methanol. m.p. 161–164 °C; IR: 3465, 3312, 1625, 1598, 1568, 1520, 1475, 1449, 1167, 1154, 1019, 948, 158, 691, 648, 526, 503, 488 cm^−1^; ^1^H NMR (500 MHz, DMSO-*d*_6_): δ 7.15–7.30 (br s, 2H, NH_2_), 7.44–7.48 (m, 3H, Ph), 7.95–7.97 (m, 2H, Ph), 8.54 (s, 1H, CH), 8.74–8.75 (m, 1H, Pyr), 8.79 (d, 1H, Pyr, *J* = 2.7 Hz), 9.40 (d, 1H, Pyr, *J* = 1.4 Hz) ppm [35].

#### 2.1.2. Synthesis of Coordination Compounds

**Figure 1:**
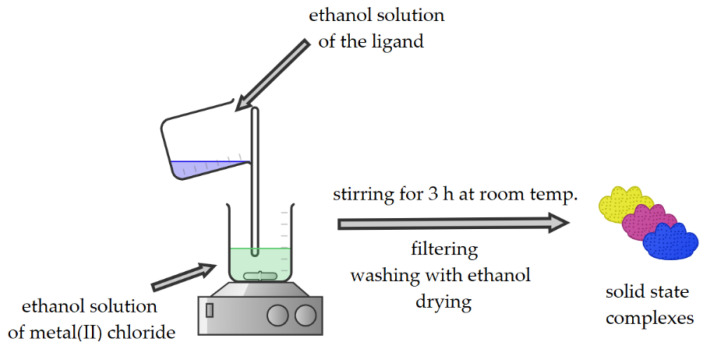
Synthesis of the investigated complexes.

In all cases, molar ratio of the ligand and copper(II), zinc(II), or cadmium(II) chloride was 1:1. Ethanol solution of the ligand was slowly added to ethanol solutions of copper(II) and zinc(II) chloride, and to water/ethanol (*v*/*v* = 1/1) solution of cadmium(II) chloride. Total volumes did not exceed 60 mL. Reaction mixtures were stirred on a magnetic stirrer for 3 h. After that time, precipitates of complexes were filtered, washed several times with ethanol, and later dried in the open air, weighted, and analyzed (Figure 1).

**Cu(L)Cl_2_**→(C_12_H_11_N_5_CuCl_2_) (359.70 g/mol) (yield: 75.2%), anal. calc. (%): Cu, 17.66; C, 40.07; H, 3.09; N, 19.47. found (%): Cu, 18.16; C, 40.01; H, 3.29; N, 19.39.
**Zn(L)Cl_2_**→(C_12_H_11_N_5_ZnCl_2_) (361.56 g/mol) (yield: 72.8%), anal. calc. (%): Zn, 18.09; C, 39.86; H, 3.07; N, 19.37. found (%): Zn, 18.80; C, 39.98; H, 3.18; N, 19.45.
**Cd(L)Cl_2_**→(C_12_H_11_N_5_CdCl_2_) (408.57 g/mol) (yield: 99.1%), anal. calc. (%): Cd, 27.51; C, 35.27; H, 2.72; N, 17.15. found (%): Cd, 26.80; C, 35.04; H, 3.00; N, 17.00.

#### 2.1.3. Formation of Silver Nanoparticles

**Figure 2:**
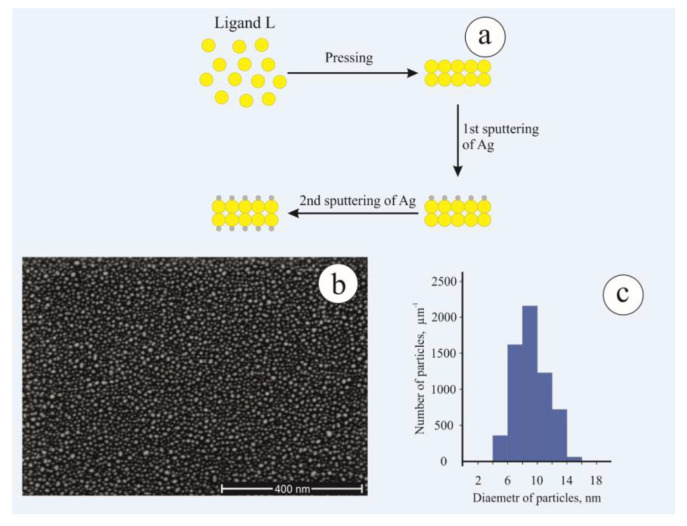
Scheme of the formation of a tablet with silver nanoparticles (**a**); SEM images (**b**) and the particles size distribution histogram (**c**) of silver nanoparticles arrays.

Pellets were made from Cu(L)Cl_2_, Zn(L)Cl_2_, Cd(L)Cl_2_ using a laboratory press AE&T T61220M. The thickness and diameter of the tablets were about 500 μm and 5 mm, respectively. The pressure applied to compress the polymer samples was in the order of 3 tons, which was applied for 5 min. At the final stage, the formation of silver nanoparticles was carried out using the method of vacuum-thermal evaporation at a residual pressure of 3 × 10^−5^ Pa. The weight of the evaporated sample of silver was about 2 mg. The distance between the substrate and the evaporated sample was about 20 cm. The evaporation was carried out on two sides of the polymer pellet (Figure 2a). The average size of silver nanoparticles was about 15–20 nm; the average height was 2–3 nm (Figure 2b,c).

### 2.2. FTIR Spectra

**Figure 3:**
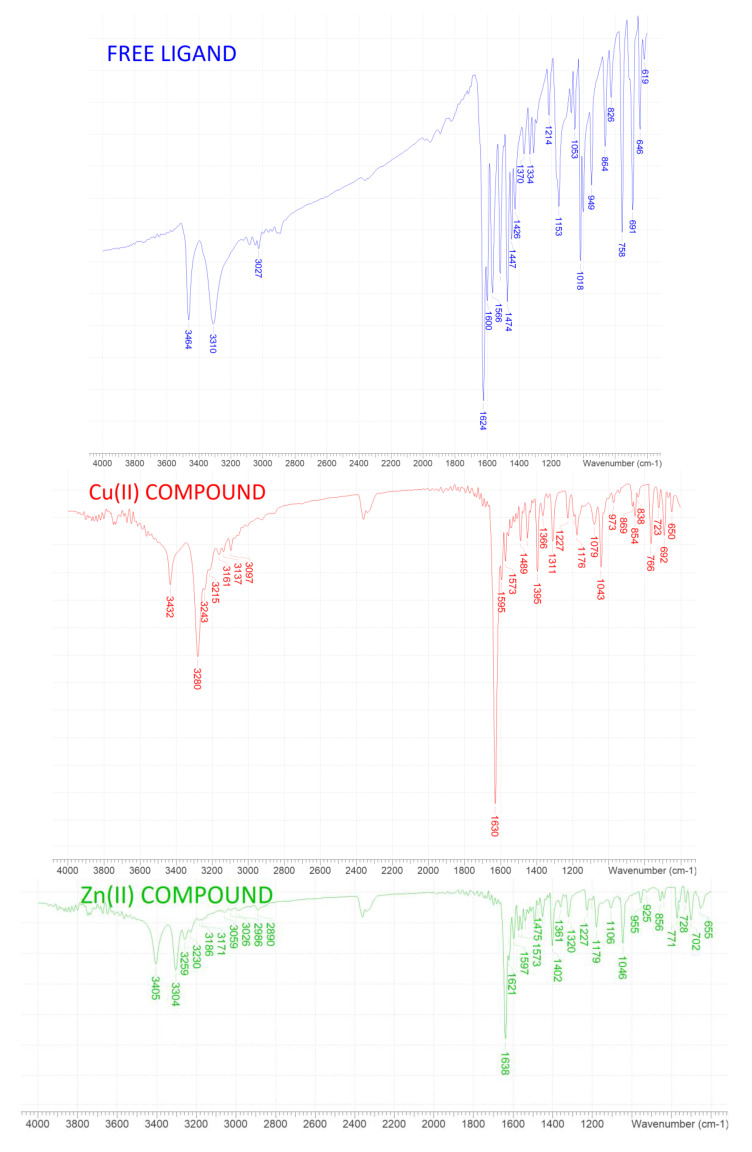

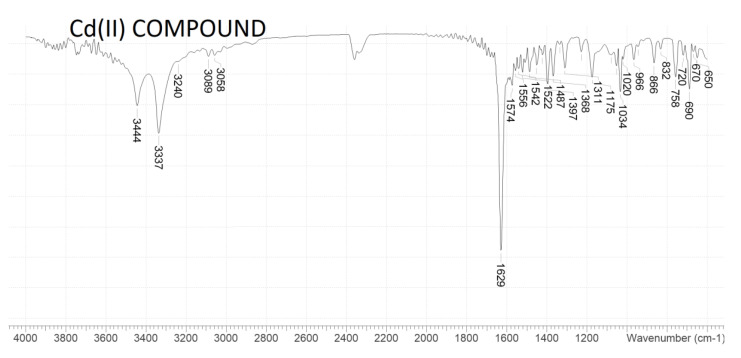
FTIR spectra of free organic ligand and studied coordination compounds.

Figure 3 shows the FTIR spectra of free ligand and studied coordination compounds. During complexation, the vibrational modes of the free ligand change. Comparing all spectra, it can be found that the fundamental ν(NH) vibrational stretching modes, which occur in free ligand at 3464 and 3310 cm^−1^, can also be observed in the spectra of the coordination compounds in the range 3444–3280 cm^−1^. The presence of NH group indicates that this group does not take part in the binding with metal ions. In the spectra of uncoordinated ligand, vibration modes of ν(C=N) and ν(C=C) appear in the ranges 1624–1566 cm^−1^ and 1520–1426 cm^−1^, respectively. As a result of coordination, these frequencies shift towards higher or lower frequencies and most of the peaks are weakened (1638–1573 cm^−1^ and 1522–1475 cm^−1^, respectively). Moving towards the lower wavelengths, we can observe bands in ranges of 1320–1034 cm^−1^ and 955–670 cm^−1^ that correspond to β(CH) and γ(CH) modes, respectively. Their lower intensity than in the free ligand results from the coordination of the metal(II) ion with two nitrogen atoms. The FTIR spectra of all complexes are very similar to each other, which is a confirmation that in all cases, organic ligand coordinates metal cations in the same manner.

### 2.3. UV–VIS–NIR

**Figure 4:**
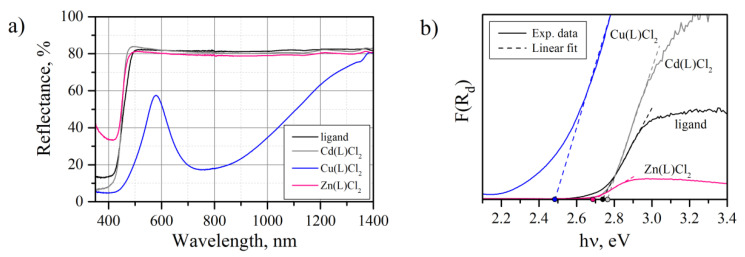
Diffuse reflectance spectra (**a**) and Kubelka–Munk plots for the optical bandgap energy calculation (**b**).

To investigate the semiconductivity of fabricated samples, the optical bandgap (E_g_) was estimated from UV–VIS–NIR spectra. The diffuse reflectance spectra of the powder samples were measured using a UV–VIS–NIR spectrophotometer Agilent Cary 5000 with an integrating sphere. The spectra are shown in Figure 4a.

The diffuse reflectance data have been transformed into an absorbance according to the Kubelka–Munk approach using F(R_d_) [36]. The equation for the Kubelka–Munk method can be represented by F(R_d_) = (1−R_d_)^2^/2R_d_, where R_d_ is the diffuse reflectance and F(R_d_) is the absorbance. It should be noted that there are several other approaches (F(R) *hv*)^n^ taking into account the type of transition in the forbidden zone [37]; however, here, we have used the classical approach without considering any type of transition.

The calculated F(R_d_) curves of the samples are shown in Figure 4b. The optical bandgap energies of all synthesized samples were estimated from the intercept of tangents drawn to the plots. The results of estimating the optical bandgaps from the experimental spectra are shown in Table 1.

The results have demonstrated that the investigated compounds are wide-bandgap semiconductors. Despite the fact that, as shown by FTIR, organic ligand coordinates the investigated metal cations in the same manner, their effect on the optical bandgap is different. If samples of Zn(L)Cl_2_ and Cd(L)Cl_2_ exhibited similar bandgap energies compared with the ligand, the Cu(L)Cl_2_ differs markedly from them.

### 2.4. Thermal Studies in Air

**Figure 5:**
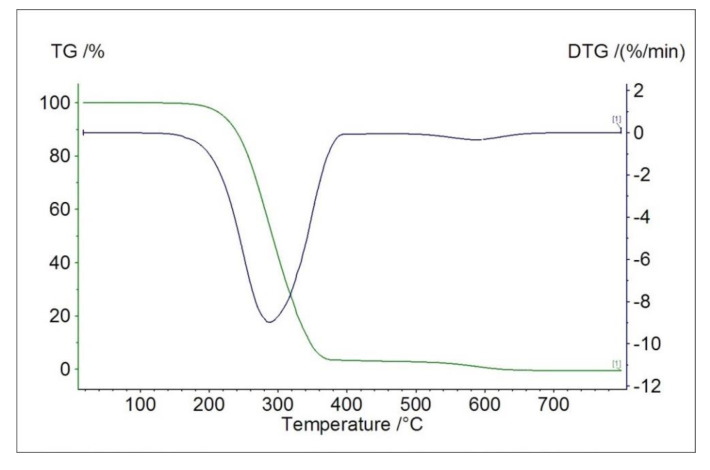
TG (green) and DTG (blue) curves showing ligand decomposition pattern in air.

Ligand itself is thermally stable up to 140 °C. Its decomposition is presented in Figure 5. From 140 °C up to 380 °C, we observe main mass loss (mass loss found, 96%). From this temperature up to 700 °C, we can observe a small mass loss (mass loss found, 4%), which can most likely be ascribed to postcombustion processes of the organic molecule.

**Figure 6:**
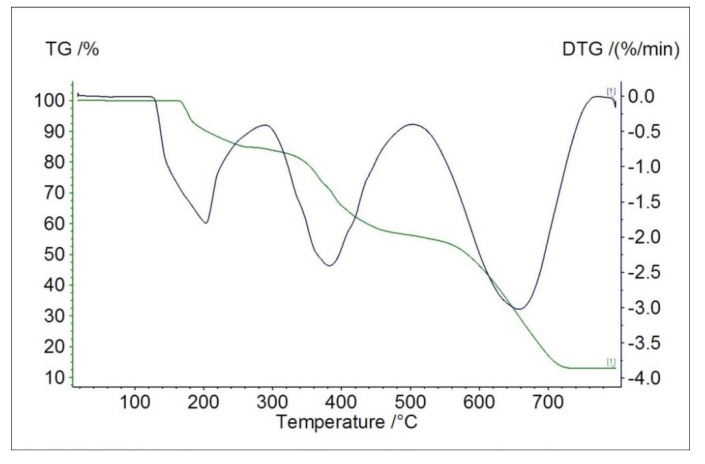
TG (green) and DTG (blue) curves showing Cu(F)Cl_2_ compound decomposition pattern in air.

Decomposition of Cu(L)Cl_2_ compound (Figure 6) begins at 160 °C. From this temperature up to 500 °C, we observe two-step, partial decomposition of the organic ligand (mass loss found, 55.5%; mass loss calculated, 55.39%). Horizontal mass level appears at 740 °C. The last mass loss is connected with postcombustion processes of the organic molecule, as well as, most likely, formation of volatile copper(I) chloride polymers, which has already been reported in the literature [38].

**Figure 7:**
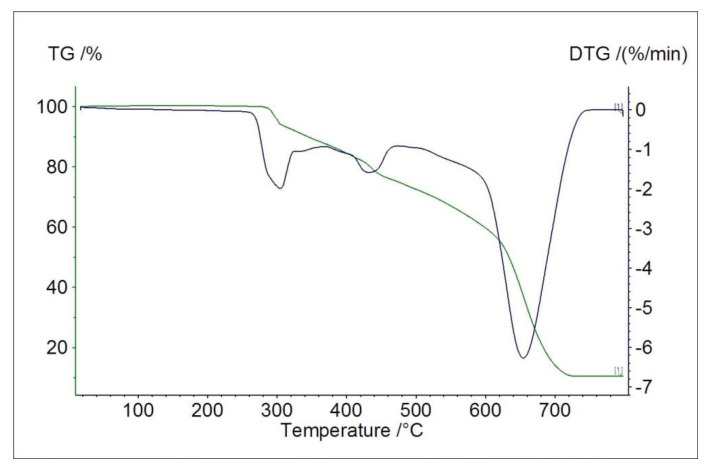
TG (green) and DTG (blue) curves showing Zn(L)Cl_2_ compound decomposition pattern in air.

Zn(L)Cl_2_ compound (Figure 7) is stable up to 260 °C. From this temperature up to 445 °C, we observe partial decomposition of the organic ligand (mass loss found, 22.0%; mass loss calculated, 21.87%). Next, up to 740 °C, we observe total thermodestruction of the organic molecule. Further in this case, volatile polymers are most likely to be formed, since horizontal mass is lower than theoretical, calculated for zinc(II) oxide.

**Figure 8:**
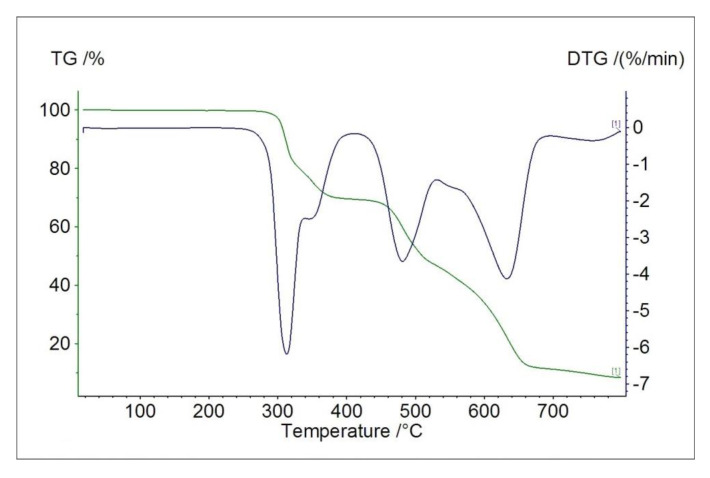
TG (green) and DTG (blue) curves showing Cd(L)Cl_2_ compound decomposition pattern in air.

Cd(L)Cl_2_ compound starts decomposing at 260 °C (Figure 8). The first part of decomposition is partial destruction of organic ligand (260–410 °C, mass loss found, 30.5%; mass loss calculated, 29.64%). Next, we observe total decomposition of the ligand. Further in this case, it is most likely that due to the formation of volatile cadmium derivatives, we observe lower horizontal mass than calculated for cadmium(II) oxide, which appears at 790 °C.

### 2.5. DSC Study

**Figure 9:**
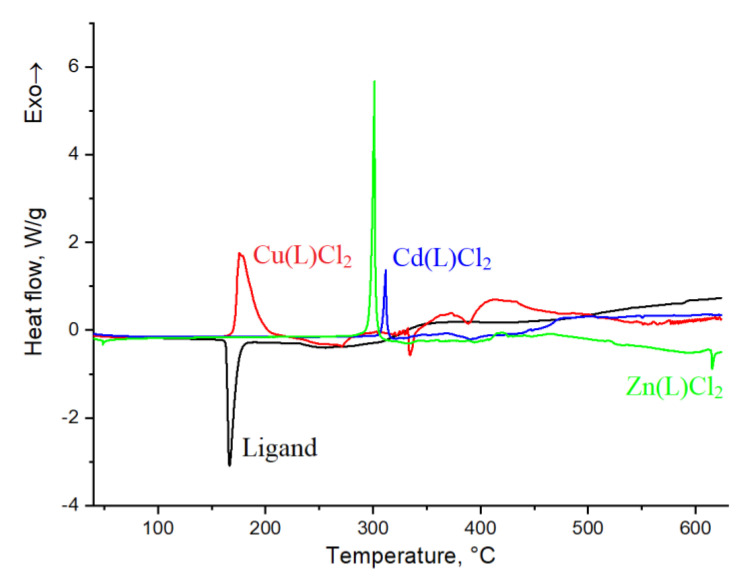
DSC scans of investigated materials.

A complex investigation of thermal parameters is a necessary step for the investigation of materials. Differential scanning calorimetry (DSC) is one of the fast and informative methods for thermal characteristics investigation. In this regard, the DSC method was used in this work. DSC scans are shown at Figure 9.

During measurements, the materials left the crucibles, violating their integrity and geometric parameters. Significant mass losses were noted after measurements. The most obvious thermal effects are shown in the Table 2.

For ligand, an endothermic melting peak is seen in the temperature range from 162 to 180 °C; this is coincident with the literature [39]. For Cu(L)Cl_2_, the appearance of additional peaks can be seen: exothermal effect from 166 to 209 °C and two endothermal effects from 332 to 343 °C and from 377 to 404 °C. For Cd(L)Cl_2_, there is an additional exothermal effect at the temperature range from 305 to 318 °C. Further, for the material with Zn(L)Cl_2_, two obvious effects can be seen: exothermal effect from 293 to 307 °C and endothermal effect from 614 to 619 °C. In addition to these thermal effects, the DSC curves also exhibit very wide exothermal effects at temperatures from 250 °C, which can be associated with decomposition processes. The appearance of additional effects on the DSC curves of doped materials can be related with the formation of new bonds and the decomposition processes. The obtained results can allow us to evaluate the effect of alloying on the properties of the material since the appearance of additional effects on the DSC curves of doped materials can be related with the formation of new bonds and the decomposition processes.

### 2.6. Magnetic Study

The superconducting quantum interference device (SQUID) is a very sensitive instrument used to characterize magnetic properties of various materials by measuring their magnetization as a function of temperature and magnetic fields. It can detect the magnetism produced by unpaired spins of electrons in radicals or by electrons of magnetic ions (typically, transition metals or rare earth elements) with a large precision. Its high sensitivity arises from the fact that the magnetic flux enclosed by any closed superconducting loop is quantized. This principle of operation currently yields the highest known experimental resolution among any other magnetic measurement techniques.

In closed shell materials (where all spins are paired), SQUID can be used to determine the concentration of magnetic impurities or free radicals in a specimen. This is performed by measuring the number of free spins in the sample and associating it with possible contaminants. The Quantum Design MPMS XL7 device used in the current experiments can detect magnetic moments above 10^−7^ emu, which corresponds to 10^13^ spins (i.e., approx. 10^−10^ mol of magnetic impurities on a given nonmagnetic material).

**Figure 10:**
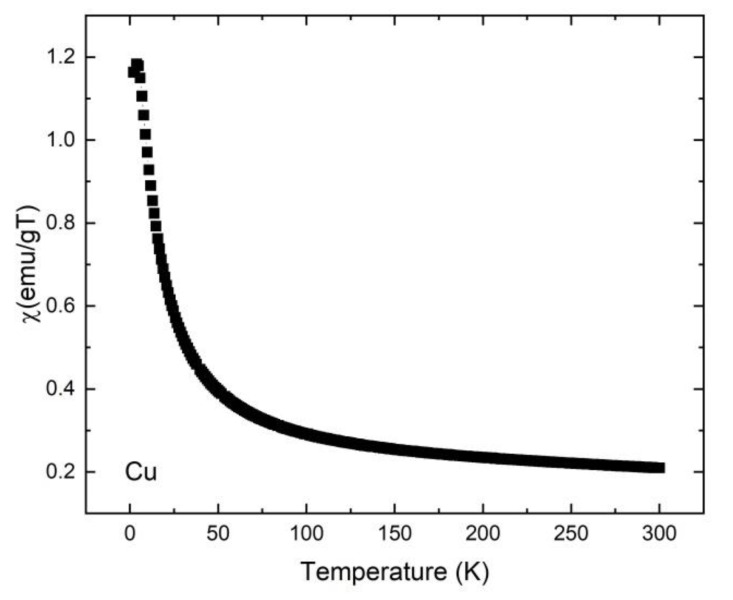
Magnetic susceptibility χ of Cu(L)Cl_2_ as the function of temperature measured at 0.6 T. Cd(L)Cl_2_ and Zn(L)Cl_2_ samples presented featureless, nearly temperature-independent curves.

**Figure 11:**
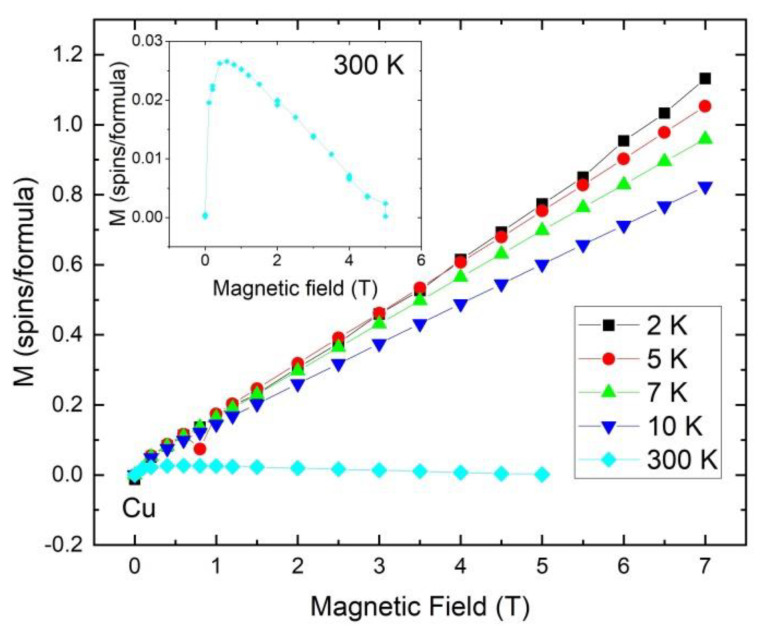
Magnetization of Cu(L)Cl_2_ as the function of magnetic field at 2.0, 5.0, 7.0, 10.0, and 300.0 K. The data show raw measurements after the extraction of an off-center linear diamagnetic background $M/B\approx -0.044\;\mu_{B}/formula\;$T^−1^, associated with the sample holder. The inset shows a magnification of the data at 300 K.

Only the complex of Cu(II) and d^9^ configuration satisfied this condition. A microcrystalline sample of Cu(L)Cl_2_ was placed in a polycarbonate capsule, which was mounted in a plastic straw. Experiments were then performed using a SQUID magnetometer (Quantum Design MPMS-XL-7T). Magnetization vs. applied field *B* curves were obtained at 2.0, 5.0, 7.0, 10.0, and 300 K, and yielded two signal sources—one associated with the sample (centered component) and another with its capsule (off-center). The magnetic susceptibility was measured as a function of temperature in heating (2 K→400 K) and cooling (400 K→2 K) modes at a rate of 1 K min^−1^ at 0.1 T (Figure 10). Results for the sample signal revealed antiferromagnetic interaction between Cu^2+^. There is no saturation of magnetization at 2.0 K—which is expected for paramagnetic, i.e., noninteracting ions. Instead, magnetization increases almost linearly at high magnetic fields (Figure 11). Such a behavior is typical for strong antiferromagnetic interactions. This suggests close contact between Cu ions. On the other hand, the sample shows weak diamagnetism at room temperature, superimposed to an equally small ferromagnetic-like background. The ferromagnetic/superparamagnetic contribution observed is consistent either with the presence of magnetite in the compound, in a proportion of 0.38% in mass, or with free radicals at the ratio of three per 100 molecules. Assuming Fe_3_O_4_ as the sole contributor to the unexpected magnetization allows for a lower limit estimation for the purity of the Cu(L)Cl_2_ compound around 99.6% in mass. The origin of such a small fraction of magnetite in the Cu(II) complex can be attributed to the handling of precursors with metal spatulas.

**Figure 12:**
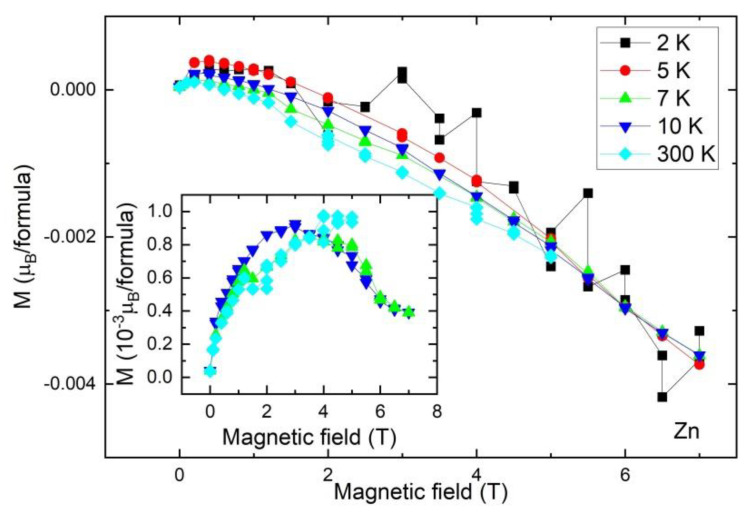
Magnetization of Zn(L)Cl_2_ as the function of magnetic field at 2.0, 5.0, 7.0, 10.0, and 300.0 K after the extraction of an off-center linear diamagnetic background $M/B\approx -0.085\;\mu_{B}/formula\;$T^−1^, associated with the sample holder. The insert shows the data for T = 7 K, 10 K, and 300 K after the subtraction of an additional diamagnetic background of $-0.04\;\mu_{B}/formula\;$T^−1^.

A microcrystalline sample of Zn(L)Cl_2_ was measured using the same procedure as described above. Unfortunately, the off-center diamagnetic signal associated with the sample holder was about one order of magnitude stronger than the sample signal, causing the diamagnetic response of the sample to be masked by the diamagnetic response of the polycarbonate capsule employed. The linear diamagnetic background was, then, estimated at $M/B\approx -0.085\;\mu_{B}/formula\;$T^−1^ based on isolated measurements of individual capsules. Using this method, the paramagnetic component of the signal, albeit noisy, could still be resolved. It presented a value at least 30 times smaller than the one observed in Cu(L)Cl_2_, allowing a lower limit estimation of magnetic impurities in this sample at 90 ppm (in mass). Results are presented in Figure 12.

**Figure 13:**
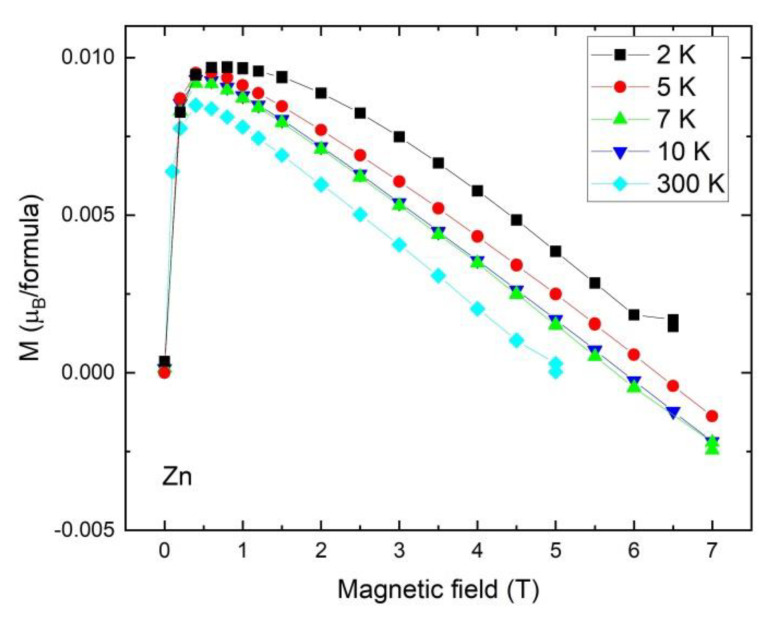
Magnetization of the Cd sample as the function of magnetic field at 2.0, 5.0, 7.0, 10.0, and 300.0 K. The data correspond to raw measurements after the extraction of an off-center linear diamagnetic background $M/B\approx -0.05\;\mu_{B}/formula\;$T^−1^, associated with the sample holder.

Lastly, a microcrystalline sample of Cd(L)Cl_2_ was characterized magnetically. Results revealed a superposition between constant diamagnetic and weakly temperature-dependent paramagnetic signals (Figure 13). The latter accounted for a single unpaired spin per 100 molecules. This value is similar to the one observed for Cu(L)Cl_2_. Assuming magnetite as the source of the magnetic signal allow us to estimate the presence of magnetic impurities at 0.025% in mass, placing a lower limit for the purity of the Cd(L)Cl_2_ complex around 99.7%.

### 2.7. MTT Cytotoxicity Assay

**Figure 14:**
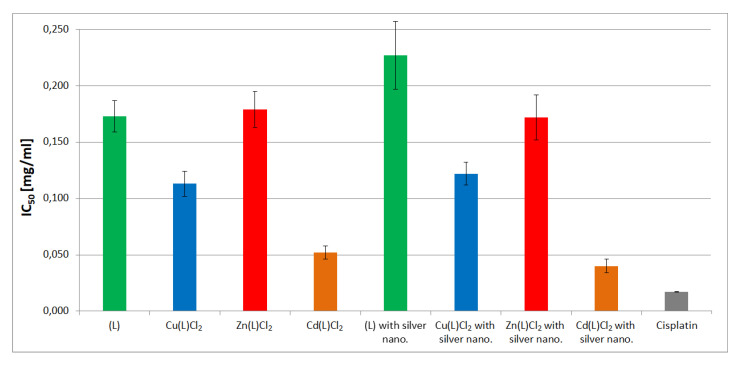
In vitro cytotoxic activity of new compounds on cancer cell lines (A549). The last bar presents IC_50_ value for cisplatin [25] as a reference. Statistical significance was assessed using one-way ANOVA analysis.

**Figure 15:**
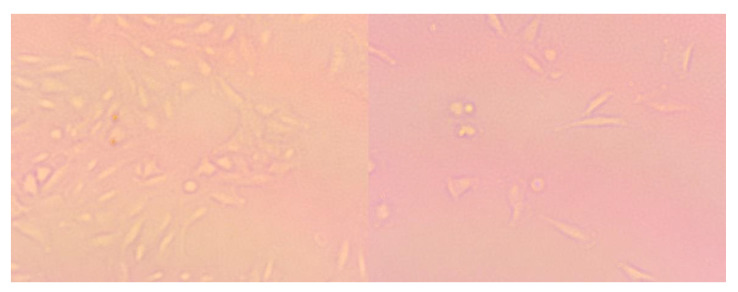
Antitumor effect of Cd(L)Cl_2_ compound with silver nanoparticles on A549 cancer cells. Cells were cultured in the presence (**right**) or in the absence (**left**) of tested compounds. Representative phase-contrast cell images are shown after 24 h (100 magnification).

In this study, the cytotoxic properties were determined using MTT cell viability assay on A549 cells. The results (Table 3, Figure 14 and Figure 15) showed that all compounds exhibited potent cytotoxic activities against A549 cell lines.

Compound Cd(L)Cl_2_ with nanosilver particles exhibited the highest cytotoxic activity with IC_50_ = 0.040 mg/mL (Figure 14). So, it can be developed for further studies as a potent antitumor agent to prevent lung cancer (Figure 15). The second most potent compound was Cd(L)Cl_2_ derivative with IC_50_ = 0.052 mg/mL. These results showed that new compounds had significant cytotoxic effects on A549 cell line, especially since they are comparable with one of the most important cytotoxic compounds: cisplatin (IC_50_ = 0.017 ± 0.0001 mg/mL) [40]. Similar results were obtained by other research teams [41,42,43]. Cadmium is a heavy metal with no physiological function and is often considered toxic [44]. After administration, Cd^2+^ ions are unable to generate free radicals directly but there is an increased production of ROS, resulting in oxidative damage to various molecules such as nucleic acid, enzymes, and membrane phospholipids [45]. This is one of the anticancer activity mechanism models of cadmium complexes. We observed that these complexes showed comparable or higher cytotoxic potential in tested A549 cancer cell line. The cadmium complexes could induce cell death through apoptosis and arrest the cells acting as cell-cycle-specific chemotherapeutic agents. It can be induced by an increase in ROS formation, which induces DNA damage and interaction with DNA repair mechanisms. Additionally, using silver nanoparticles, we increased the safety of using metal complex [46].

### 2.8. ADME Analysis

ADME (Absorption, Distribution, Metabolism, and Excretion) studies are performed to characterize the molecules of a new compound before starting biological studies. Characterization of ADME properties helps to explore how pharmacokinetic processes happen. The ADME analysis was performed using the SWISSADME platform (Swiss Institute of Bioinformatics 2021) [47,48,49]. SwissADME enables calculation of the physicochemical, pharmacokinetic, and drug-likeness of new compounds. Toxicity prediction was performed using ProTOX II service [47].

**Figure 16:**
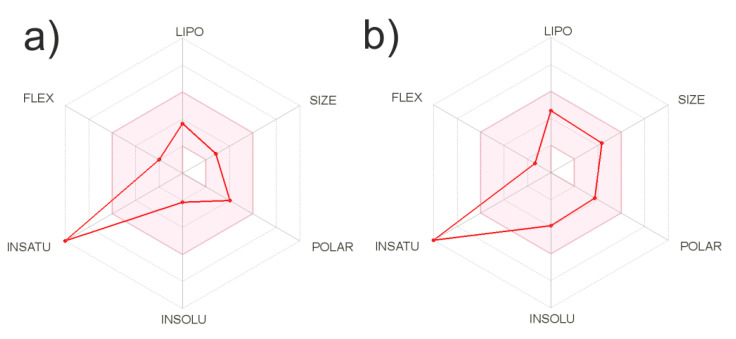
Bioavailability radar (lipophilicity (LIPO) are within the range −0.7 < XlogP3 < +5.0; molecular weight (SIZE) are 150 g/mol < MW < 500 g/mol; polarity (POLAR) are 20 Å² < TPSA < 130 Å²; insolubility (INSOLU) are 0 < logS < 6;—insaturation (INSATU) are 0.25 < Fraction Csp3 < 1; Flexibility (FLEX) are 0 < Num. rotatable bonds < 9) for ligand (**a**) and complex (**b**).

**Figure 17:**
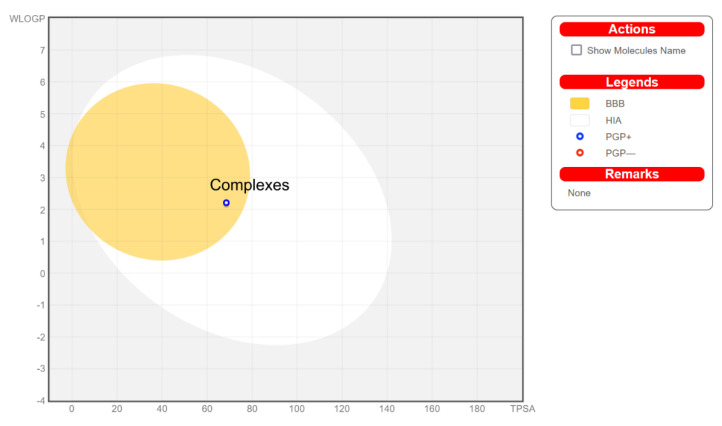
BOILED-Egg diagram for all compounds.

Performing an ADME analysis allows only those compounds that exhibit high biological activity and low toxicity to be selected for testing. Determination of drug-likeness, the chance of a compound becoming an oral drug, was performed by analyzing the physicochemical and structural properties. A bioavailability radar was generated for the ligand (Figure 16a) and one of the complexes (all complexes have similar plots—Figure 16b). All substances have satisfactory ADME properties, and the plots suggest high bioavailability; the only unfavorable property was a high unsaturation score in compounds. It is, however, important to notice that both ligand and all tested complexes meet the rules of Lipinski [50], Ghose [51], Egan [52], Veber [53], and Muegge [54]. Therefore, it can be concluded that all tested compounds are good drug candidates. Gastrointestinal absorption and brain access are important in selecting compounds for biological research and drug discovery. The BOILED-Egg diagram (Figure 17) shows that the complexes penetrate the blood–brain barrier and are absorbed gastrointestinally. The tested compounds were selected due to their physicochemical parameters, which indicate that they will be good candidates in the search for new active drugs.

Servis ProTox II classified ligand to toxicity class 4 (harmful if swallowed (300 < LD_50_ ≤ 2000)); predicted LD_50_, 400 mg/kg). All complexes belong to the same toxicity class (predicted LD_50_, 1000 mg/kg for Cu(L)Cl_2_ and Cd(L)Cl_2_; LD_50_, 825 mg/kg for Zn(L)Cl_2_. The ligand showed good results on toxicity radar: 0.56 probability of being carcinogenic, 0.68 probability of hepatotoxicity, and 0.57 probability of mutagenicity.

## 3. Materials and Methods

### 3.1. Chemistry

All of the chemicals used for the synthesis were purchased from Sigma-Aldrich, AlfaAesar, and POCH and were used without further purification. The contents of Cu(II), Zn(II), and Cd(II) in solid complexes were determined by the F-AAS spectrometer with a continuum source of light and using air/acetylene flame. Absorbance was measured at analytical spectral lines; limit of quantification was 0.04 mg/L. Solid samples were decomposed using the Anton Paar Multiwave 3000 closed system instrument. Mineralization was carried out for 45 min at 240 °C under pressure 60 bar. The contents of carbon, hydrogen, and nitrogen were determined by a Vario microcompany Elementar Analysensysteme GmbH. The FTIR spectra were recorded with an IRTracer-100 Schimadzu Spectrometer (4000–600 cm^−1^), with a recording accuracy of 1 cm^−1^ using KBr pellets. Reflectance spectra were measured in the range from 250 to 1900 nm (the measuring limit of the instrument was 2600 nm) in 1 nm steps using an Agilent Cary 5000 spectrophotometer at room temperature. The spectra were measured using an integrating sphere with a diameter of 110 mm. The studied powders were mechanically rubbed into filter paper. A similar filter paper from the same batch, but without the rubbed-in powder, was used as a standard set in the path of the second beam of the spectrophotometer. The morphology of silver nanoparticles was studied using a Thermo Scientific Quattro SEM scanning electron microscope. The thermolysis of the compounds in the air atmosphere was studied using TG–DTG technique in the temperature range of 25–800 °C at a heating rate of 10 °C min^−1^; TG and DTG curves were recorded on a Netzsch TG 209 apparatus under air atmosphere (v = 20 mL min^−1^) using ceramic crucibles. Ceramic crucibles were also used as a reference material. Thermal properties were investigated by differential scanning calorimetry (DSC-50, Shimadzu) at a heating rate of 10 °C min^−1^ in a nitrogen flow (v = 20 mL min^−1^) from ambient temperature up to 630 °C. The powder samples had masses of a few milligrams and were pressed in Al pans. Empty Al pans were used as references. Temperature calibration was checked with In, Sn, Pb, Cd, and Zn for chosen heating rate.

### 3.2. ADMET Analysis

Free ligand and coordination compounds were analyzed using ACDLabs Percepta software version 14.0.0 (Advanced Chemistry Development, Inc. Metropolitan Toronto, ON, Canada), SwissADME service (Swiss Institute of Bioinformatics, Lausanne, Switzerland, 2021) [47], admetSAR 2.0 service (admetSAR 2019) [55], and ProTOX II service [56] to obtain the computational pharmacokinetic and toxicologic profiles of the tested compounds.

### 3.3. Biological Assays

#### 3.3.1. Cell Culture

Put the A549 Cell Line human from human lung (carcinoma) supplied by European Collection of Authenticated Cell Cultures (ECACC) in Dulbecco’s Modified Eagle’s Medium (DMEM, PAN-Biotech) containing 10% of fetal bovine serum (FBS, Sigma Aldrich), 100 U/mL penicillin, and 100 μg/mL streptomycin under a humidified atmosphere containing 5% (*v*/*v*) CO_2_ at 37 °C.

#### 3.3.2. Cytotoxicity Assay

The (3-(4,5-dimethylthiazol-2-yl)-2,5-diphenyltetrazolium bromide (MTT) assay was used to evaluate the cytotoxic effect of new compounds on A549 cells as previously described [57]. A total 100 mL of DMEM fresh medium containing 10^4^ cells/well of cells was inoculated into a 96-well plate. This was cultured in an incubator for 24 h and treated with new compounds, which were dissolved in pure water or in water solution of DMSO (0.1%). After 24 h, MTT reagent (0.5 mg/mL) was added into each well, then cells were kept in the dark for 2 h to form formazan crystals. After that, the media were removed, and 100 μL DMSO was added to dissolve formazan crystals. At last, the absorbance was measured at 570 nm by Synergy H1 microplate reader (BioTek, Winooski, VT, USA). Cell viability inhibition rate was calculated and the data were expressed as mean ± SD values from three independent replicates.

## 4. Conclusions

All four compounds—ligand L and its three coordination compounds—form solids stable at room temperature. Incorporated analytical techniques—NMR spectroscopy, F-AAS spectrometry, elemental analysis, and FTIR spectroscopy—provide a good insight into their structures. It was found by UV-VIS-NIR spectrometry that the modification of ligand leads to a change in the optical properties and the width of the optical bandgap. Despite the fact that, as shown by FTIR, organic ligand coordinates the investigated metal cations in the same manner, their effect on the optical bandgap is different. If samples of Zn(L)Cl_2_ and Cd(L)Cl_2_ exhibited similar bandgap energies compared with the ligand, the Cu(L)Cl_2_ differs markedly from them. Thermogravimetric analysis of investigated complexes prove that, when heated, these compounds decompose gradually and form metal(II) oxides as the main products of decomposition. DSC measurements allowed us to identify and investigate the most important endo- and exothermic effects connected with the decomposition process. The DSC results showed that the modification with complexes leads to the appearance of additional thermal effects, which is associated with the breaking of new bonds or decomposition processes. Magnetic studies revealed strong antiferromagnetic interaction and, thus, proximity of Cu(II) ions in Cu(L)Cl_2_. A lack of appreciable positive components and transitions in the magnetic susceptibility of Zn(L)Cl_2_ and Cd(L)Cl_2_ above 0.5 T, on the other hand, suggest closed-shell compounds with weak intermolecular interactions.

Results of ADME analysis for all compounds—ligand and its complexes—indicate that they are good candidates for novel drugs and that the compounds satisfy the druglike conditions. All tested compounds are absorbed from the gastrointestinal tract. Additionally, the examined complexes cross the blood–brain barrier.

Both the ligand and its coordination compounds exhibit strong cytotoxic activity against A549 cancer cells. Of all investigated compounds, cadmium complex with silver nanoparticles showed the highest activity (IC_50_ = 0.040 ± 0.006 mg/mL) and can be considered a promising candidate for further exploration as an anticancer agent. This is a very good result, especially when compared with one of the most important cytotoxic compounds—cisplatin (IC_50_ = 0.017 ± 0.0001 mg/mL) [25].

Usually, Ag^+^ can be taken up by the cells. Then, ions enter the cytosol through ion transporters [58]. The small nanoparticles we presented can interact with the surface of the cells (plasma membranes). In our case, nanoparticles, as active nanocomplexes, can penetrate the cell membrane and accumulate in internal cell compartments. The mechanisms for capturing nanoparticles in cells were likely phagocytosis, endocytosis, or micropinocytosis. The effect of this mechanism is a cytotoxic effect on neoplastic cells [59,60,61]. Research so far indicates that silver nanoparticles can lead to damage in almost every part of the cell, including mitochondria [62], nucleus [63], and endoplasmic reticulum [64]. Moreover, coordination compounds themselves often show very promising anticancer properties. The exact mechanism, however, remains unknown [65]. Research indicates that they can work through various mechanisms, such as inhibition of proteasome activity, telomerase activity, formation of reactive oxygen species (ROS), DNA degradation, DNA intercalation, and paraptosis [66,67]. The theory of the reactive oxygen species and the induction of oxidative stress is also important. This applies to both nanoparticles and coordination compounds [62]. Their relative importance in the toxicity of both types of Ag substances has not been studied [68]. There are still many challenges to understand how the silver nanoparticles work. In this case, it is important to provide more research in this area.

Further modifications of ligand may lead to enhancement of its anticancer properties. Silver is one of the most important molecules, widely known for its folk biocidal properties [69]. Many research teams report the extraordinary biological properties of silver nanoparticles. First of all, it relates to their toxicity effect against bacterial and cancer cells [70]. The medicaments currently used as an anticancer treatment are toxic to the body, producing side effects and unintended or untargeted effects on normal body physiology. Silver nanoparticles exhibit promising results in anticancer therapy. Application of this type of metal provides an increase in the safety of new molecules compared with conventional therapy. Drugs currently used as anticancer medicines are toxic to the body, have side effects, and have unintended or untargeted influence on the proper physiology of the organism. Nanoparticles provide safe and effective therapeutic agents in the treatment of cancer. When formulated with different metals such as Cu, Zn, and Cd they can exhibit different properties and activities; therefore, they are expected to exhibit different mechanisms of action such as interaction with DNA, induction of apoptosis, or inhibition of protein. In this way, new anticancer agents can be obtained [71].

## Figures and Tables

**Table 1:** Optical bandgaps (E_g_, eV) from the experimental spectra for ligand and studied complexes.

Sample	Ligand	Cu(L)Cl_2_	Zn(L)Cl_2_	Cd(L)Cl_2_
E_g_, eV	2.74	2.48	2.69	2.76

**Table 2:** Mass loss and thermal effects in investigated samples.

Material	Mass Loss after 1st Measurement/%	Thermal Effect/°C			
		Type	Onset	Max	Endset
L	98.6	Endo	162.2	166.6	179.8
Cu(L)Cl_2_	50.0	Exo	165.5	176.0	209.4
		Endo	332.0	334.5	343.3
		Endo	376.7	388.0	404.2
Zn(L)Cl_2_	33.2	Exo	293.3	301.6	306.7
		Endo	614.4	615.4	618.5
Cd(L)Cl_2_	56.3	Exo	304.8	311.5	318.4

**Table 3:** In vitro cytotoxic activity of new compounds on cancer cell lines (A549). All values are presented as mean ± standard deviation (SD). IC_50_—50% inhibition of the cell viability, mg/mL.

Compound	IC_50_ (mg/mL)
Ligand (L)	0.173 ± 0.014
Cu(L)Cl_2_	0.113 ± 0.011
Zn(L)Cl_2_	0.179 ± 0.016
Cd(L)Cl_2_	0.052 ± 0.006
Ligand (L) with silver nanoparticles	0.227 ± 0.030
Cu(L)Cl_2_ with silver nanoparticles	0.122 ± 0.010
Zn(L)Cl_2_ with silver nanoparticles	0.172 ± 0.020
Cd(L)Cl_2_ with silver nanoparticles	0.040 ± 0.006
